# Optimization of Seed Oil Extraction from *Asphodelus tenuifolius* Cav. Using Response Surface Methodology

**DOI:** 10.3390/plants14152298

**Published:** 2025-07-25

**Authors:** Fatima Ezzahra Eddaoudi, Chakir El Guezzane, Hamza El Moudden, Ayoub Badi, Yousra El Idrissi, Hicham Harhar, Agnese Santanatoglia, Filippo Maggi, Giovanni Caprioli, Abdelhakim Bouyahya, Mohamed Tabyaoui

**Affiliations:** 1 Laboratory of Materials, Nanotechnology and Environment, Faculty of Science, Mohammed V University of Rabat, Rabat BP 1014, Morocco; fatieddaoudi84@gmail.com (F.E.E.); chakir.elguezzane@gmail.com (C.E.G.); ayoub_badi@live.fr (A.B.); yousra.elidrissi93@gmail.com (Y.E.I.); h.harhar@um5r.ac.ma (H.H.); h.tabyaoui@um5r.ac.ma (M.T.); 2 Laboratory of Applied Sciences for Sustainable Development, Higher School of Technology of El Kelaa Des Sraghna, Cadi Ayyad University, Marrakech BP 104, Morocco; h.elmoudden@uca.ac.ma; 3 School of Pharmacy, Chemistry Interdisciplinary Project (ChIP), University of Camerino, Via Madonna delle Carceri s.n.c., 62032 Camerino, Italy; agnese.santanatoglia@unicam.it (A.S.); filippo.maggi@unicam.it (F.M.); 4 Laboratory of Human Pathologies Biology, Department of Biology, Faculty of Sciences, Mohammed V University in Rabat, Rabat BP 1014, Morocco; a.bouyahya@um5r.ac.ma

**Keywords:** *Asphodelus tenuifolius*, Soxhlet extraction, response surface methodology (RSM), fatty acid composition, Linoleic acid

## Abstract

Two solvents, *n*-hexane and ethyl acetate, were employed to extract oil from *Asphodelus tenuifolius* Cav. seeds using the Soxhlet extraction technique. The process was optimized using Central Composite Design (CCD) and Response Surface Methodology (RSM). ANOVA and a second-order polynomial equation were applied to evaluate the effects of key operational factors, including extraction time (20–60 min) and solvent-to-solid ratio (0.2–0.6 g/mL), on oil yield. The physicochemical properties, fatty acid composition, and functional groups of the extracted oil were analyzed. While both solvents influenced oil yield and quality, the fatty acid composition remained consistent, with unsaturated fatty acids, particularly linoleic acid, identified as the main components. Under optimized conditions, the highest oil yields were 22% with *n*-hexane and 19.91% with ethyl acetate. FTIR spectroscopy confirmed the presence of ester groups, suggesting potential applications in biodiesel production. These findings offer valuable insights for producing oils rich in unsaturated fatty acids for food, cosmetic and renewable energy industries. These findings pave the way for further advancements in industrial applications by promoting the sustainable use of plant-derived oils.

## 1. Introduction

*Asphodelus tenuifolius* Cav., commonly known as onion weed, belongs to the Asphodelaceae family. Native to the Mediterranean region, it is now also cultivated in the United States, Malaysia, Australia, Chile, New Zealand and Mexico. Traditionally, this species has been used as a diuretic and for treating various ailments, including colds, rheumatic pain, hemorrhoids and inflammatory disorders [1]. The fixed oil extracted from *A. tenuifolius* seeds is highly valued for its medicinal and therapeutic properties, primarily due to its rich linoleic acid content, which has potential benefits in preventing arteriosclerosis [2]. Various extraction techniques have been employed to obtain oil from *A. tenuifolius* seeds, including Soxhlet extraction, ultrasonic extraction, and microwave-assisted extraction. Among these, Soxhlet extraction has proven to be the most efficient [3]. The efficiency of the extraction process is influenced by several factors, such as solvent type, extraction time, particle size, temperature, and solid-to-solvent ratio. To optimize the extraction process, advanced statistical approaches such as Design of Experiments (DoE) and Response Surface Methodology (RSM) are often employed. These techniques enable the simultaneous analysis of multiple variables to determine optimal conditions. In particular, RSM and Central Composite Design (CCD) are valuable tools for optimizing complex processes. RSM not only assesses the individual impact of variables but also explores their interactions, providing a comprehensive understanding of the operational parameters [4]. This study aims to enhance the conditions for Soxhlet extraction of *A. tenuifolius* seed oil using Response Surface Methodology. Additionally, the extracted oils were analyzed, and their fatty acid compositions were determined.

## 2. Materials and Methods

### 2.1. Materials

*A. tenuifolius* seeds were manually collected in southern Morocco in April 2024. The seeds were left to dry in the shade at room temperature and subsequently ground using a mixer grinder without additional preparation. All chemicals and solvents used in the study were of analytical grade and purchased from Merck and Sigma-Aldrich (Steinheim, Germany).

### 2.2. Extraction Procedure

The solvents used for oil extraction from the samples were n-hexane and ethyl acetate. The powdered *A. tenuifolius* seeds were placed in a Soxhlet extractor, and the solvent was added to the round-bottom flask of the apparatus. Subsequently, 200 mL of the solvent was introduced and heated to the temperatures specified in the experimental design (68 °C for n-hexane and 77 °C for ethyl acetate). The seeds were extracted multiple times over durations ranging from 20 to 60 min, using different solid-to-solvent ratios (0.2–0.6 g/mL). Each experiment was conducted in triplicate, and the average yield was calculated to validate the optimal conditions predicted by the model.

The oil yield was determined gravimetrically:

$$\mathrm{yield}\;=\frac{\mathrm{weight}\;\mathrm{of}\;\mathrm{extracted}\;\mathrm{oil}\;\times 100\%}{\mathrm{weight}\;\mathrm{of}\;\mathrm{dry}\;\mathrm{seeds}}$$

### 2.3. Experimental Design

Analysis of Variance (ANOVA) was combined with Central Composite Design (CCD) and Response Surface Methodology (RSM) to optimize the oil extraction yield (Y). Two independent variables were considered for this optimization: solvent-to-solid ratio (X_1_) and extraction time (X_2_). These variables were tested at three levels, low (−1), medium (0) and high (+1), as detailed in Table 1. An empirical model was constructed to describe the relationship between the extraction yield (Y) and the operational variables. This model employs a second-order polynomial equation to account for the effects of the independent variables.

(1) $$Y=\gamma_{0}+\sum \gamma_{i}X_{i}+\sum \gamma_{ii}X_{i}^{2}+\sum \gamma_{ij}{X_{i}X}_{j}$$

*Y* is the response; γ_0_ (constant), γ_i_ (linear), γ_ii_ (quadratic) and γ_ij_ (interactive) are the regression coefficients; and X_i_ and X_j_ are the independent variables.

### 2.4. Characterization of Extracted Oils

The quality of the oils extracted using n-hexane and ethyl acetate was evaluated using standard methods, including acid value, iodine value and saponification value [5]. The fatty acid composition and spectral characteristics of the oils obtained from *A. tenuifolius* were analyzed through gas chromatography (GC) and Fourier Transform Infrared (FT-IR) spectroscopy, as detailed in this study. Gas chromatography Varian CP-3800 (Varian Inc., Walnut Creek, CA, USA) was used with a flame ionization detector (FID) and a CP-Wax 52CB column (30 m × 0.25 mm). A flow rate of 1 mL/min of helium was used as the carrier gas. After being initially set at 170 °C for one minute, the oven temperature was raised to 230 °C at a rate of 4 °C per minute. The total GC analysis time was 26 min. Fatty acids were identified by comparing the retention times of the chromatographic peaks obtained with those in the instrument’s library. The results were expressed as a relative percentage of the area of each fatty acid peak compared to the total area. The Fourier Transform Infrared (FTIR) spectrometer (Bruker Corporation, Billerica, MA, USA) was employed to record spectra with a resolution of 4 cm^−1^ in the 4000–400 cm^−1^ range. Two pellets of potassium bromide (KBr) were placed between the oil samples. Origin 8.0 software was used for spectral analysis.

## 3. Results

### 3.1. Extraction Process Modeling and Optimization

The experimental data obtained from the oil extraction process, including the observed results and predicted values, are summarized in Table 2. A quadratic regression model was employed to analyze this data, and its validity was assessed using Analysis of Variance (ANOVA).

For n-hexane:

Y_H_ = 19.269286 + 2.641667R − 2.0083333T + 0.525RT − 0.738571RR + 0.648571TT (2)

For ethyl acetate:

Y_E_ = 15.585714 + 3.766667R − 1.2266667T + 0.4RT − 0.101429RR + 0.171429TT (3)

where Y is the percentage oil extracted; R, and T are the uncoded independent variable values, namely solvent ratio and extraction time.

ANOVA results for the Central Composite Design are presented in Table 3a,b. Fisher’s F-test yielded values of 9.0922 (hexane) and 354.6237 (ethyl acetate) for the model, accompanied by significant probability values (*p* < 0.05), confirming the significance of the model. Additionally, the coefficients of determination (r^2^) for the two solvents were 0.919128 and 0.997749, while the adjusted coefficients of determination (r_adj_^2^) were 0.818039 and 0.994936, respectively, indicating that the model effectively captures the combination of all considered factors. According to [6], a determination coefficient exceeding 0.75 suggests that the model is adequate. The main effects, namely solvent ratio (R) and extraction time (T), significantly influenced oil yields for both hexane and ethyl acetate (*p* < 0.05). However, the quadratic effects of RR, TT and RT on hexane yield, as well as RR and TT on ethyl acetate yield, were not significant (*p* > 0.05), implying primarily linear relationships between these variables and the respective yields within the tested range.

**Figure 1:**
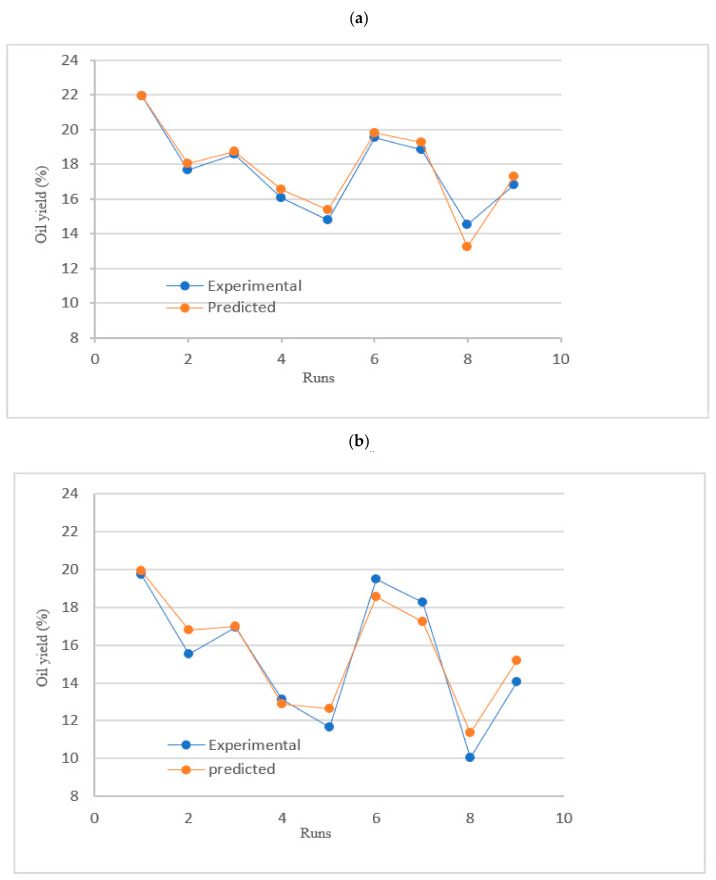
Predicted vs. experimental oil yields (%) for *A. tenuifolius* seed extraction using (**a**) n-hexane and (**b**) ethyl acetate.

The plot shows that the experimental values are evenly distributed around the predicted values, further validating the model’s accuracy (Figure 1). This alignment is consistent with previous studies emphasizing the importance of residual analysis in model validation [7,8]. The observed pattern supports the use of multiple regression analysis for exploring the relationships between independent variables and the response, as documented in various works on Response Surface Methodology and optimization techniques [7,9]. Predictive model validity has been broadly confirmed by validation methods such as goodness-of-fit and residual analysis [10]. The alignment of predicted and observed values confirms the accuracy of the applied regression models.

**Figure 2:**
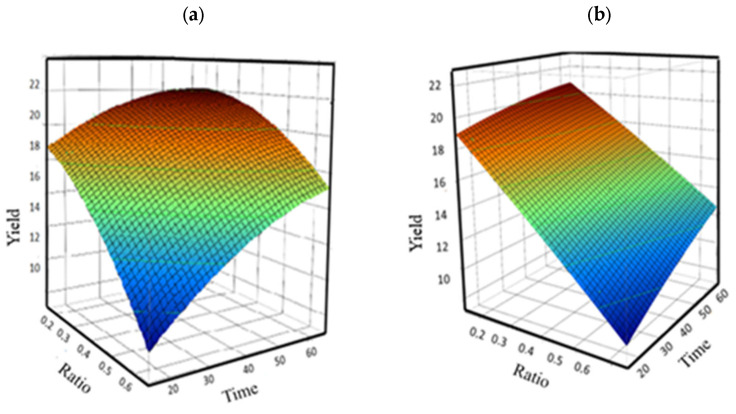
Three-dimensional surface plots illustrating the effects of extraction time and solid-to-solvent ratio on oil yield using (**a**) *n*-hexane and (**b**) ethyl acetate.

Figure 2 illustrates the effects of two parameters on oil yield using a 3D surface plot. For both *n*-hexane and ethyl acetate, a negative correlation was observed between the solvent-to-solid ratio and extraction time on oil yield. Specifically, oil yield increased with longer extraction times and lower solvent-to-solid ratios.

**Figure 3:**
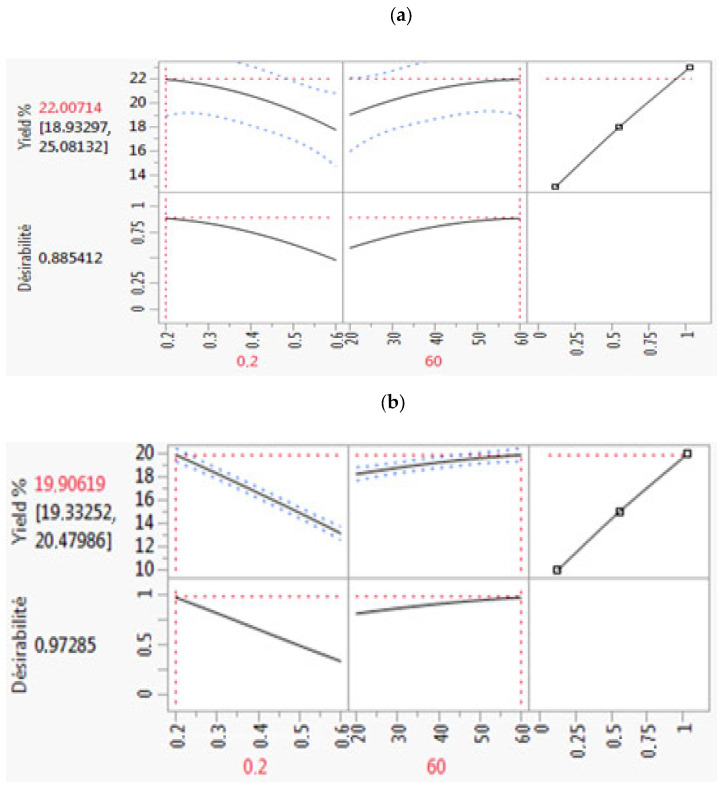
Optimization of extraction conditions for maximum oil yield using desirability function analysis: (**a**) n-hexane and (**b**) ethyl acetate. The plots indicate the optimal parameter settings for maximizing yield.

This result is likely due to deeper solvent penetration into the *A. tenuifolius* powder. A lower solvent-to-solid ratio increases the surface area, allowing better solvent diffusion into the material, thereby improving oil extraction efficiency [11,12]. To enhance the extraction process and maximize oil yield, desirability function optimization was applied. This statistical technique evaluates multiple variables simultaneously to identify optimal conditions. As shown in Figure 3, the optimal extraction parameters were achieved with a solvent system of *n*-hexane and ethyl acetate, an extraction time of 60 min, and a solid-to-solvent ratio of 0.2 g/mL. Under these conditions, the estimated oil yields were 22.00714% for *n*-hexane and 19.90619% for ethyl acetate. The desirability function values approached 1, demonstrating the successful optimization of the oil extraction process using Response Surface Methodology (RSM) [13].

### 3.2. Characterization of the Extracted Oil

The properties of the extracted oil samples were assessed (Table 4).

The oil yield obtained with *n*-hexane (21.95 ± 0.05%) was higher than that with ethyl acetate (19.91 ± 0.01%). This indicates that *n*-hexane is a more efficient solvent for extracting oil from *A. tenuifolius* seeds, likely due to its non-polar nature, which better dissolves the non-polar lipids present in the seeds. The oil extracted with *n*-hexane was golden yellow, while the oil obtained with ethyl acetate appeared brown at room temperature. This color difference may result from the higher content of unsaturated fatty acids in the more polar ethyl acetate samples. Similar trends have been observed in Kariya oil extracted using acetone, *n*-hexane, and ethyl acetate, where the polarity of ethyl acetate influenced oil properties [14]. Additionally, the oil extracted with ethyl acetate exhibited higher acid values compared to *n*-hexane, likely due to its higher polarity. The iodine value, which measures the degree of unsaturation (C=C bonds), was also higher for the ethyl acetate extract compared to the *n*-hexane extract [15]. High iodine values indicate elevated levels of unsaturated fatty acids, which may lead to faster oxidation if the oil is not stored properly.

The fatty acid composition of the oils in this study was compared with previously published data (Table 5). All the oils analyzed contained higher levels of unsaturated fatty acids compared to saturated fatty acids, with linoleic acid being the predominant component [16,17].

For both solvents, the percentage of fatty acids in the oil extracted from *A. tenuifolius* seeds was similar. This is consistent with previous studies, suggesting that solvent choice has minimal impact on the fatty acid composition of the oil [11]. The data in Table 5 reveal a predominance of polyunsaturated fatty acids (PUFAs), representing almost 79% of the total fatty acids present in the oils studied, a value higher than those reported in the literature [16,17]. This composition is particularly interesting from a nutritional point of view, as it is now well established that a diet rich in PUFAs and low in saturated fatty acids (SFAs) is beneficial to health, particularly for the prevention of cardiovascular disease [18]. This high proportion of PUFAs is explained in particular by the high linoleic acid content, which exceeds that of other fatty acids and is in line with the observations made for red bell pepper seed oils reported by [19].

**Figure 4:**
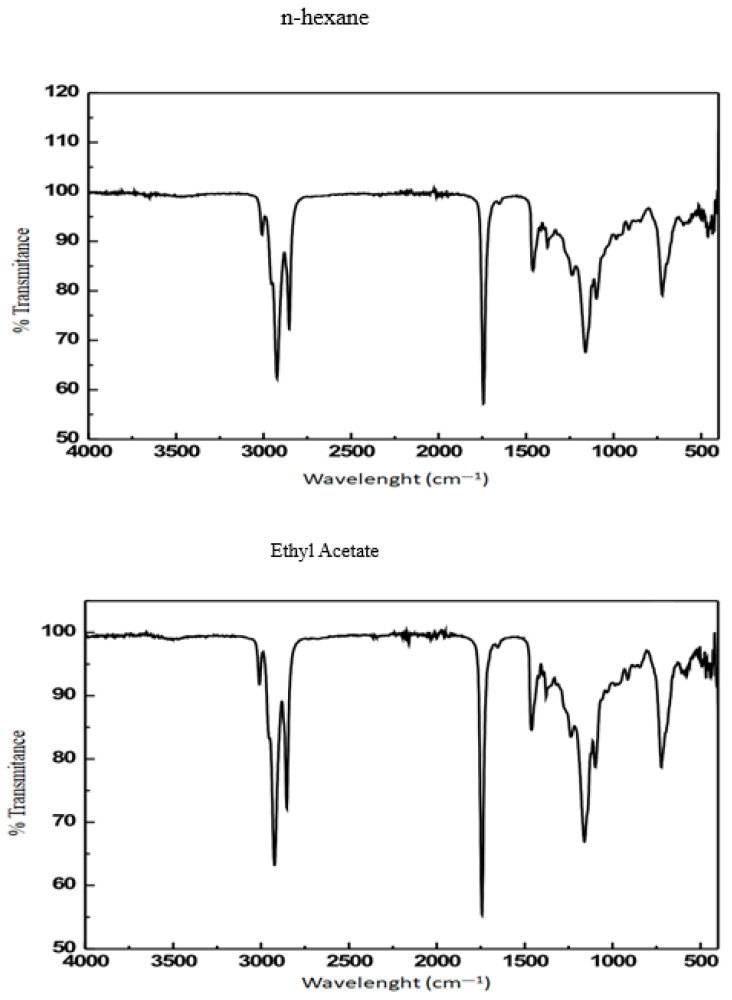
FTIR spectra of *A. tenuifolius* seed oil extracted using n-hexane and ethyl acetate.

Figure 4 and Table 6 summarize the FTIR spectral characteristics of the seed oil, highlighting the identification of functional groups. The spectra for *n*-hexane and ethyl acetate extracts were comparable, indicating no significant difference between the two solvents. This observation aligns with previous studies on solvent-based oil extraction [20]. Absorption bands at 1743 cm^−1^ and 1162 cm^−1^ confirmed the presence of ester groups, characterized by C=O and C-O stretching vibrations, respectively [21]. These bands are associated with triglycerides and their derivatives, suggesting that the extracted oil could potentially be processed into new esters [22,23]. This finding suggests that the oil has potential applications as a low-grade raw material for biodiesel production, consistent with research on biodiesel derived from non-edible oils [24,25].

## 4. Conclusions

The optimization of Soxhlet extraction for *A. tenuifolius* seed oil using two distinct solvents demonstrates the efficiency of Central Composite Design (CCD) and Response Surface Methodology (RSM) in enhancing both oil yield and quality. This study underscores the critical influence of solvent-to-solid ratio and extraction time on extraction efficiency. *n*-Hexane proved to be more effective for shorter extraction durations, whereas ethyl acetate, despite requiring longer extraction times, achieved comparable yields. The high linoleic acid content, as well as the presence of triglycerides in the extracted oil, underline its potential for exploitation in various sectors. These results indicate that this oil is a promising raw material for use in the food, cosmetics and pharmaceutical industries.

## Figures and Tables

**Table 1:** Experimental design matrix for Soxhlet extraction of *A. tenuifolius* seed oil, detailing the levels of independent variables (solid-to-solvent ratio and extraction time).

Independent Variables	Symbol		Coded Level		
	Coded	Uncoded	−1	0	+1
Ratio (g/mL)	X_1_	R	0.2	0.4	0.6
Time (min)	X_2_	T	20	40	60

**Table 2:** Experimental responses for oil extraction yield (%) from *A. tenuifolius* seeds using *n*-hexane and ethyl acetate, including observed and predicted values from Response Surface Methodology (RSM).

No. of Run	Solid/Solvent Ratio (mg/mL)	Extraction Time (min)	Coded X_1_	Level X_2_	% Oil Yield for Hexane	Predicted % Oil Yield	% Oil Yield for Ethyl Acetate	Predicted % Oil Yield
1	0.2	60	−1	+1	21.96	21.91	19.70	19.93
2	0.4	40	0	0	17.65	18.03	15.52	16.77
3	0.4	60	0	+1	18.59	18.75	16.91	16.99
4	0.6	60	+1	+1	16.05	16.55	13.11	12.86
5	0.6	40	+1	0	14.78	15.36	11.65	12.64
6	0.2	40	−1	0	19.53	19.79	19.47	18.53
7	0.2	20	−1	−1	18.86	19.25	18.24	17.23
8	0.6	20	+1	−1	14.51	13.25	10.05	11.34
9	0.4	20	0	−1	16.81	17.27	14.04	15.18

**Table 3:** (**a**) ANOVA results for the regression model of oil extraction using *n*-hexane, showing statistical significance for the tested variables and model adequacy. (**b**) ANOVA results for the regression model of oil extraction using ethyl acetate, confirming significant effects of extraction parameters on oil yield.

(**a**)					
**Source**	**Sum of Square**	**df**	**Mean Square**	**F-Value**	***p*-Value**
Model	69.87	5	13.9751	9.0922	0.0263 *
R	41.87	1	41.870417	27.2410	0.0064 *
T	24.20	1	24.200417	15.7449	0.0166 *
RT	1.102	1	1.102500	0.7173	0.4447
RR	1.27	1	1.272805	0.8281	0.4143
TT	0.98	1	0.981505	0.6386	0.4690
Residual	6.15	4	14.16		
Pure Error	0.00005	1	0.00005		
Total Error	6.15	4			
r^2^	0.919128				
r _adj_^2^	0.818039				
(**b**)					
**Source**	**Sum of Square**	**df**	**Mean Square**	**F-Value**	***p*-Value**
Model	94.904065	5	18.9808	354.6237	<0.0001 *
R	85.126667	1	85.126667	1590.445	<0.0001 *
T	9.028267	1	9.028267	168.6776	0.0002 *
RT	0.640000	1	0.640000	11.9573	0.0259 *
RR	0.024005	1	0.024005	0.4485	0.5297
TT	0.068571	1	0.068571	1.2811	0.3209
Residual	0.214095	4	0.0535		
Pure Error	0.00020000	1	0.000200		
Total Error	0.21409524	4			
r^2^	0.997749				
r _adj_^2^	0.994936				

* Significant at *p*-Value < 0.05.

**Table 4:** Physicochemical properties of *A. tenuifolius* seed oil extracted using *n*-hexane and ethyl acetate, including yield, acid value, saponification index, and iodine value.

Parameters		
Solvent	Hexane	Ethyl Acetate
Yield (%)	21.95 ± 0.05 ^a^	19.91 ± 0.01 ^b^
Physical state at 25 °C	Golden yellow	Dark brown
Acid value (mg KOH/g oil)	1.95 ± 0.00 ^a^	4.88 ± 0.00 ^b^
Saponification index (mg KOH/oil)	200.62 ± 0.02 ^a^	222.43 ± 0.00 ^b^
Iodine index (g I_2_/100 g oil)	168.87 ± 0.00 ^a^	176.96 ± 0.00 ^b^

Data are presented as the means of two single replicates (n = 2 ± SEM); (a,b) different letters within a row indicate significant statistical differences (*p* ≤ 0.05).

**Table 5:** Fatty acid composition of *A. tenuifolius* seed oil extracted using n-hexane and ethyl acetate, compared with previous literature data.

Fatty Acids	This Study		Malmir et al. (2018) [16]	Bassam (2013) [17]
	Hexane	Ethyl Acetate	NR	NR
**Saturated**				
Myristic (C14:0)	0.05 ± 0.00 ^a^	0.04 ± 0.00 ^a^	3.96	-
Palmitic (C16:0)	6.86 ± 0.01 ^a^	6.41 ± 0.00 ^b^	13.84	7.60
Heptadecanoic (C17:0)	0.06 ± 0.00 ^a^	0.09 ± 0.00 ^b^	-	-
Stearic (C18:0)	2.20 ± 0.10 ^a^	2.33 ± 0.01 ^a^	-	16.0
Arachidic (C20:0)	0.10 ± 0.00 ^a^	0.08 ± 0.00 ^b^	-	-
**Monounsaturated**				
Palmitoleic (C16:1)	0.08 ± 0.00 ^a^	0.07 ± 0.00 ^b^	-	-
Heptadecenoic (C17:1)	0.02 ± 0.00 ^a^	0.01 ± 0.00 ^a^	-	-
Oleic (C18:1)	11.66 ± 0.00 ^a^	11.79 ± 0.01 ^b^	15.60	12.80
Gadoleic (C20:1)	0.20 ± 0.00 ^a^	0.18 ± 0.00 ^b^	-	-
**Polyunsaturated**				
Linoleic (C18:2)	78.50 ± 0.00 ^a^	78.91 ± 0.00 ^b^	62.62	78
Linolenic (C18:3)	0.07 ± 0.00 ^a^	0.06 ± 0.00 ^a^	2.60	-
**SFA**	9.27 ± 0.12 ^a^	8.96 ± 0.00 ^a^	17.80	23.60
**MUFA**	11.97 ± 0.00 ^a^	12.06 ± 0.00 ^a^	15.60	12.80
**PUFA**	78.57 ± 0.01 ^a^	78.97 ± 0.01 ^b^	65.22	78

Data are presented as the mean of two single replicates (n = 2 ± SEM). (a,b) different letters within a row indicate significant statistical differences (*p* ≤ 0.05).

**Table 6:** Functional groups identified in *A. tenuifolius* seed oil using FTIR spectroscopy, highlighting key absorption bands and their corresponding molecular vibrations.

Wavenumber (cm^−1^)	Functional Group	Vibration	Intensity
2919–2854	C–H	Stretching of methyl group	Strong
1743	–C=O	Stretching of esters	Strong and sharp
1464	–C-H	Bending vibration of CH2	Variable
1162	C–O	Stretching of carboxylic acids, esters	Variable
725	=C–H	CH out-of-plane deformation	Strong

## Data Availability

All data are available in this manuscript.
